# Necrotizing Pancreatitis After Bland Embolization of a Large Hepatic Hemangioma

**DOI:** 10.14309/crj.0000000000001471

**Published:** 2024-08-22

**Authors:** Ayesha Khan, Julia Hawes, Julia Zhang, Ahmed Khan, Karen Szauter, Maryamnaz Falamaki, Michael L. Kueht, Luca Cicalese, Sreeram Parupudi

**Affiliations:** 1 Internal Medicine, University of Texas at Medical Branch, Galveston, TX; 2 University of Texas at Medical Branch SOM, Galveston, TX; 3 Department of Gastroenterology and Hepatology, University of Texas at Medical Branch, Galveston, TX; 4 Department of Radiology, University of Texas at Medical Branch, Galveston, TX; 5 Department of Surgery, University of Texas at Medical Branch, Galveston, TX

**Keywords:** necrotizing pancreatitis, post embolization syndrome, liver embolization

## Abstract

Liver embolization is a common procedure for management of liver lesions. Embolization can be performed using only an embolic material or along with chemotherapy agents. Infrequent complications seen postliver embolization include pulmonary thromboembolism, hepatic infarct, liver abscess, liver failure, ischemic biliary strictures, and less frequently pancreatic damage (incidence of 1.7%). We describe a case of necrotizing pancreatitis after bland embolization of a large hepatic hemangioma. The exact mechanisms of acute pancreatitis after liver embolization are uncertain, although direct ischemic mechanisms, toxic effects of antineoplastic agents, and volume of embospheres used are believed to play a role.

## INTRODUCTION

Liver embolization is a frequently performed procedure for the management of liver lesions.^1^ Embolization can be performed through a bland technique using only an embolic material or through chemotherapy agents, namely, transarterial chemoembolization. In cases of benign lesions such as hepatic hemangiomas, bland embolization is an appropriate treatment option that is utilized.^2^ The most common condition after embolization is postembolization syndrome consisting of fever, abdominal pain, nausea, and vomiting.^3^ Other infrequent complications seen postliver embolization (bland and transarterial chemoembolization) are pulmonary thromboembolism, hepatic infarct, liver abscess, liver failure, ischemic biliary strictures, and less frequently pancreatic damage.^1,4^ We report a case of necrotizing pancreatitis after bland embolization of a large hepatic hemangioma.

## CASE REPORT

**Figure 1.**
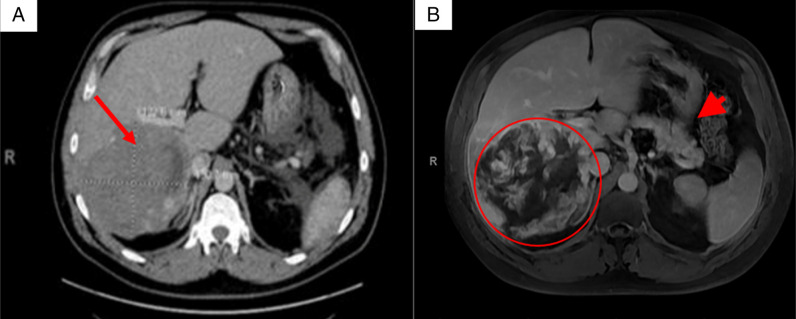
(A) Lobulated, heterogeneous density lesion seen in the right lobe liver, most compatible with a hemangioma. (B) Magnetic resonance imaging axial section T1 fat saturated postcontrast during the venous phase: An 18 cm right hepatic lobe mass with incomplete peripheral nodular enhancement characteristic for hemangioma (circle). A normal pancreas is partially seen (short arrow).

**Figure 2.**
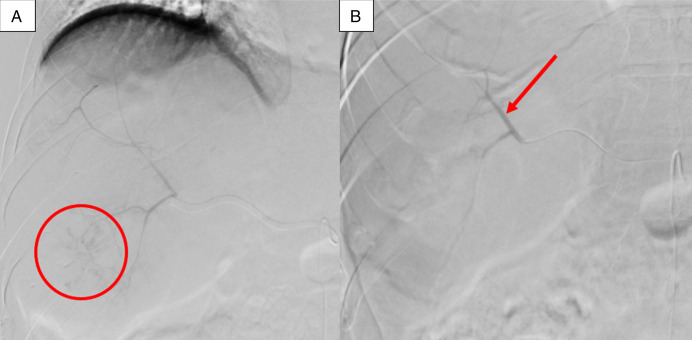
(A) Scattered nodular blush in the inferior segment of right hepatic lobe in the territory of posterior division of the right hepatic artery without active contrast extravasation or pseudoaneurysm (circle). (B) Successful particle embolization of the posterior division of right hepatic artery with 400 μm microspheres demonstrating near complete stasis of the antegrade flow (arrow).

**Figure 3.**
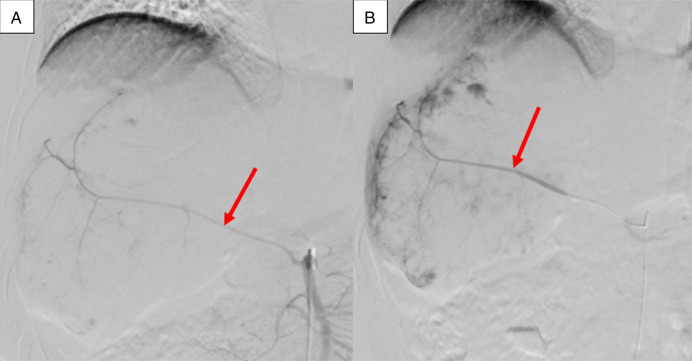
(A) Superior mesenteric arteriogram showing a dominant accessory right hepatic artery (arrow). Extensive nodular tumor blush is noted in the right liver lobe with no active extravasation or pseudoaneurysm, consistent with known hepatic hemangioma. (B) Successful particle embolization of the accessory right hepatic artery with 400 μm and 500 μm microspheres demonstrating complete stasis of the antegrade flow into the treated artery (arrow).

**Figure 4.**
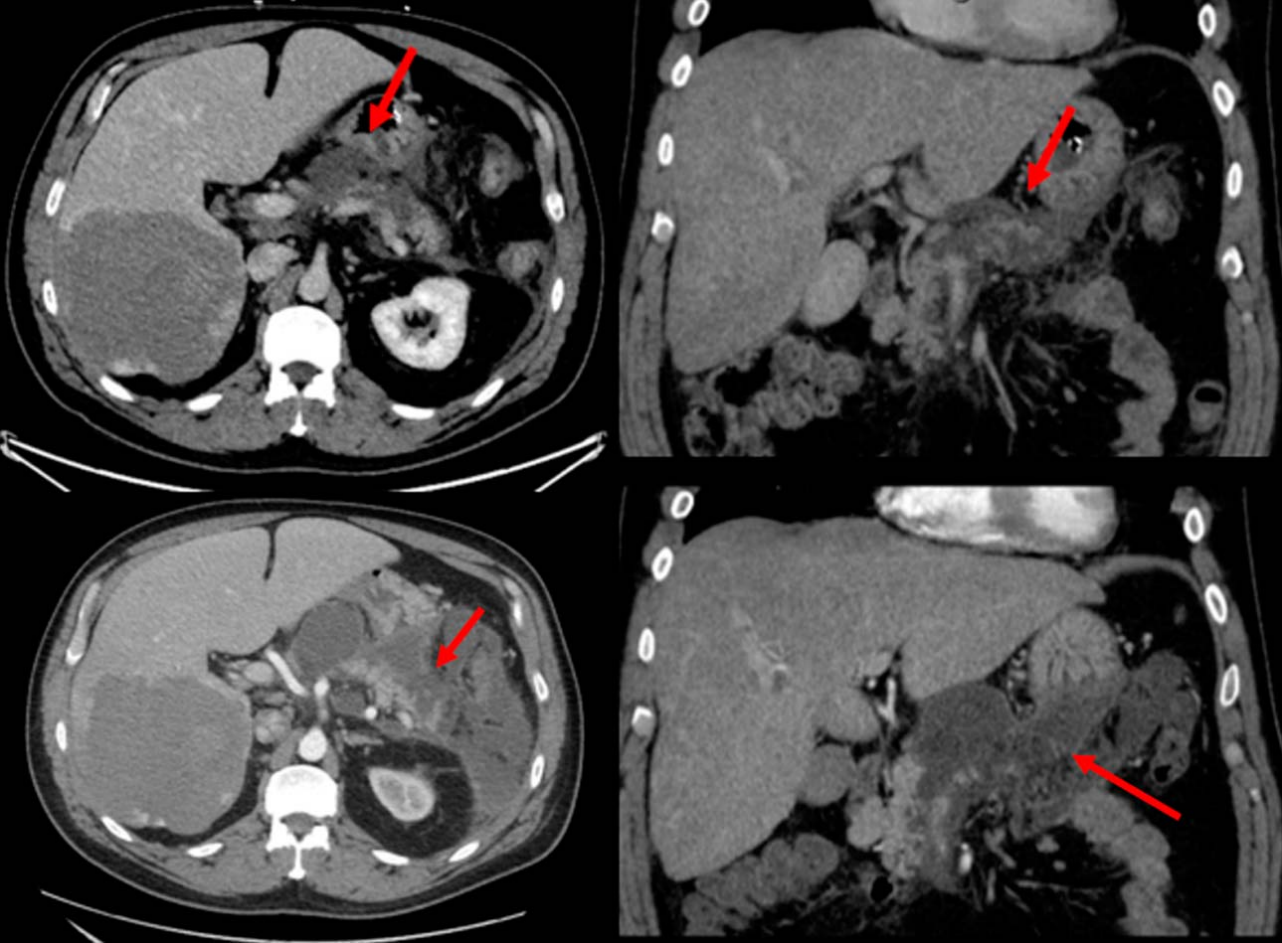
Axial and coronal postcontrast computed tomography scan—two days after liver embolization showing pancreatic necrosis and surrounding edema (arrow) [top image]. Worsening pancreatic necrosis and peripancreatic collections (arrows) 7 days after embolization [bottom image].

A 47-year-old White man with a medical history of class I obesity with a body mass index of 31.2 kg/m^2^, controlled hypertension on atenolol, hyperlipidemia on simvastatin, and stable generalized anxiety disorder on escitalopram presented for worsening right upper quadrant pain and tenderness for 2 weeks. His pain was worse postprandially and described as constant, sharp, and tender. He had a 20-pack year history of tobacco smoking without any alcohol or drug use. On evaluation, the patient's vital signs were stable, and physical examination demonstrated a palpable mass in his right upper abdomen. Admission laboratory studies showed a mildly elevated alkaline phosphatase of 154 U/L. The remaining liver profile, lipase, complete blood count, and basic metabolic panel were normal. An abdominal computed tomography (CT) scan revealed a 15.3 cm right hepatic lobe mass consistent with a hemangioma (Figure 1). Magnetic resonance imagining of the abdomen characterized the mass at 18 cm (Figure 1). Periprocedural arteriograms confirmed a large hemangioma supplied by the proper right hepatic artery (originating from celiac artery) and the accessory right hepatic artery (originating from superior mesenteric artery). In preparation for hepatic resection, the arteries were successfully embolized by 400 and 500 μm microsphere particles, using 4 mL of embospheres under real-time ultrasound guidance. The procedure was deemed successful assessed through postembolization angiography with the superior mesenteric artery demonstrating complete stasis of the antegrade flow into the treated artery, and the patient had no periprocedural complications (Figures 2 and 3). The following day, the patient developed acute-onset nausea with bilious vomiting, epigastric pain, and fever (up to 101 F). His white blood cell (WBC) count increased from 7,000 WBCs/μL before the embolization to 18,000 WBCsla/μL the following day. Two-day postprocedure, a CT scan with contrast was obtained demonstrating acute necrotizing pancreatitis involving a large volume of the pancreas without acute necrotic collection with no signs of gallstones and normal bile duct anatomy; his amylase was 408 U/L and lipase was 951 U/L (normal reference values are 35–110 and 0–220, for amylase and lipase, respectively) (Figure 4). Triglyceride levels within normal limits. The patient had no history of pancreatitis or alcohol abuse. He was transferred to the surgical intensive care unit; empiric antibiotics and total parenteral nutrition were started. Repeat CT abdomen findings after 7 days showed irregularly marginated peripancreatic fluid collections. With clinical improvement, the patient was discharged with plans for follow-up and future hemangioma resection.

**Figure 5.**
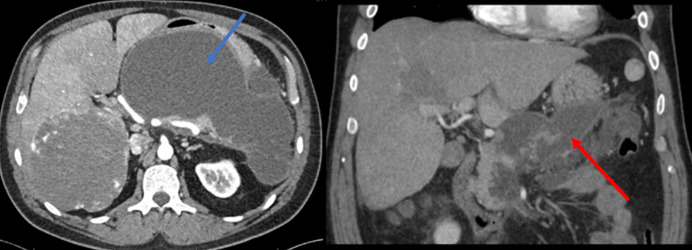
Axial and coronal postcontrast computed tomography scan. Thirty-three days after embolization. Multiple pancreatic pseudocysts. The largest pseudocyst now measures 22.3 ×10.5 × 10 cm (blue arrow) and development multiloculated peripancreatic fluid collections/walled off necrosis (red arrow).

**Figure 6.**
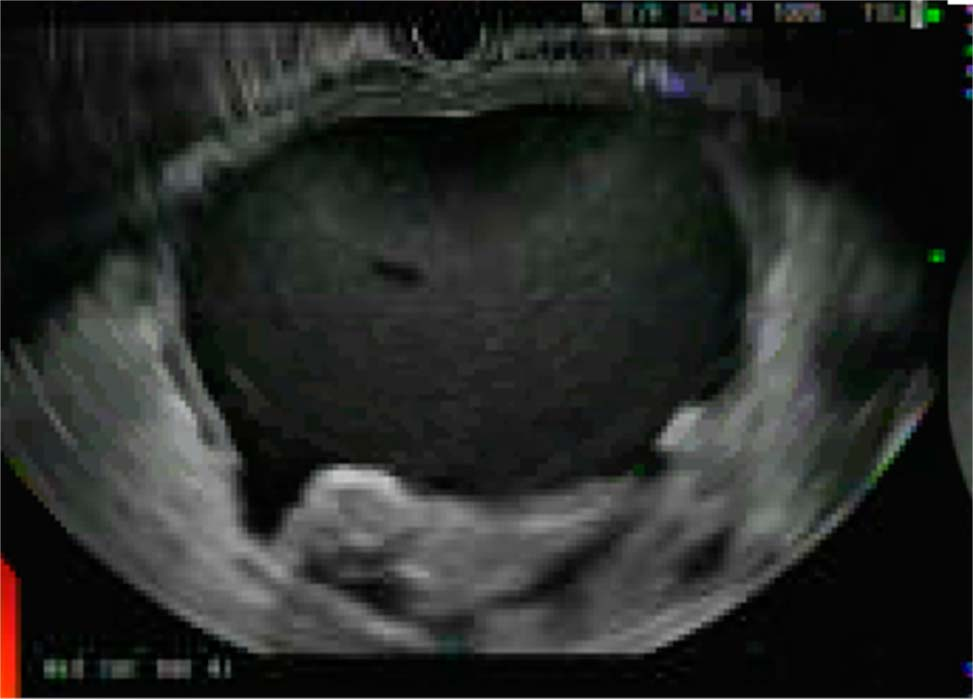
90 mm × 100 mm peripancreatic fluid collection.

**Figure 7.**
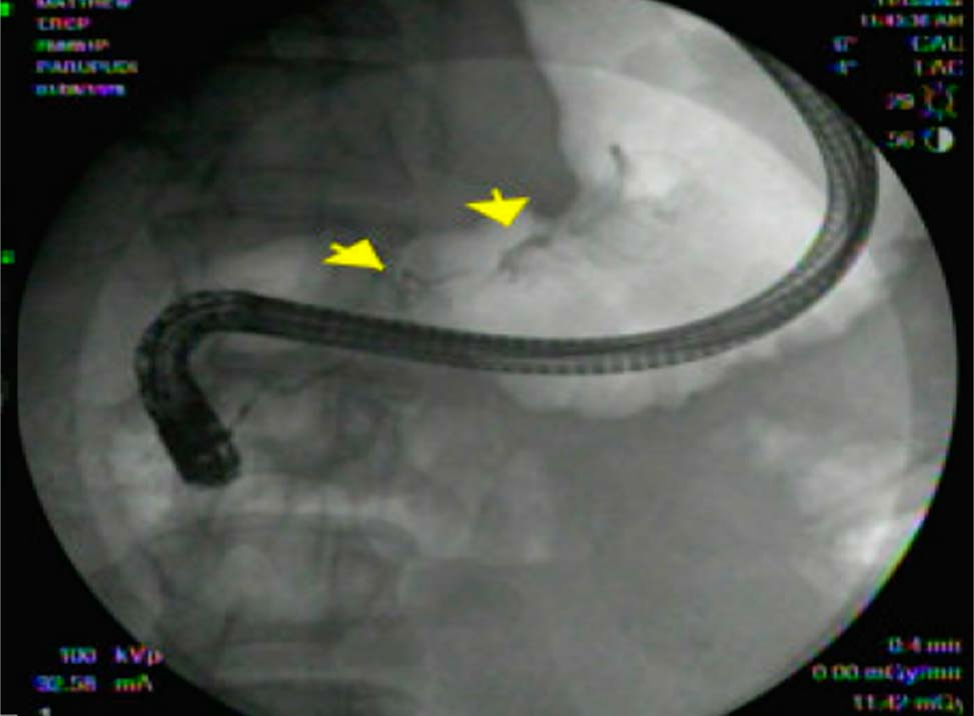
Pancreatogram showing ductal disruption (arrows).

**Figure 8.**
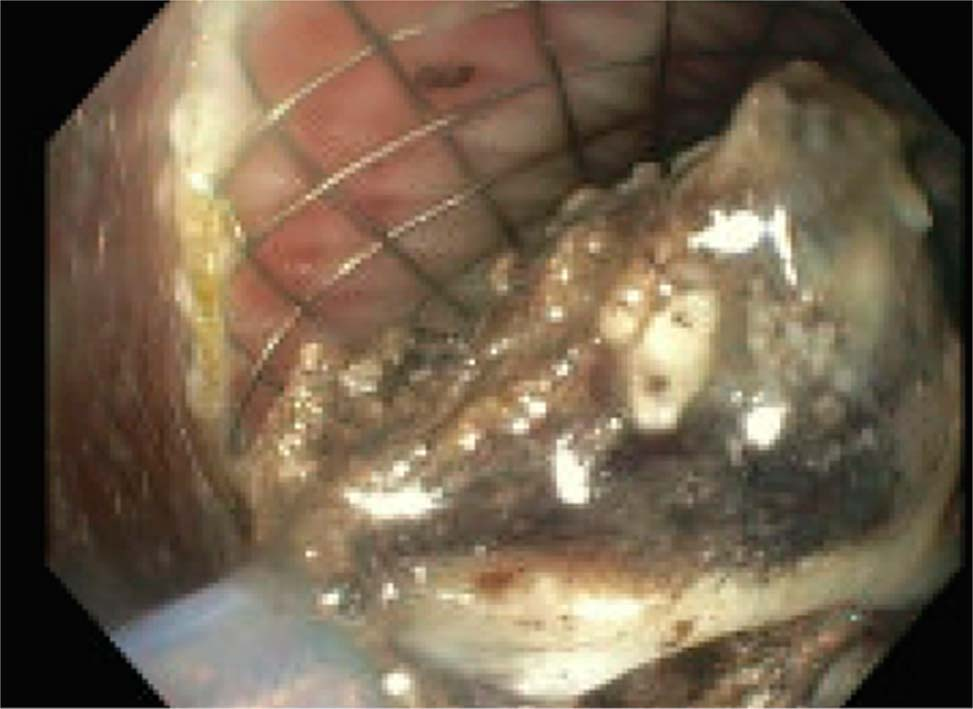
Endoscopic necrosectomy through cystogastrostomy stent showing black necrotic debris.

He was readmitted a month later with symptoms of persistent fevers up to 100.4°F, epigastric and periumbilical abdominal pain and poor appetite. A pancreatic pseudocyst per CT findings showed multiple small pseudocysts with the largest pseudocyst measuring 22.3 × 10.5 × 10 cm and development of multiloculated peripancreatic fluid collections/walled off necrosis (Figure 5). Endoscopic ultrasound was performed showing a 90 mm × 100 mm pseudocyst seen in the pancreatic body, and cystogastrostomy was done using luminal apposing metal stent—axial stent (Figure 6). A 900 cc of brown fluid drained. Three weeks later, the patient was readmitted because of repeat fevers. With a diagnosis of infected pseudocyst, an endoscopic necrosectomy was performed. To assess for pancreatic duct disruption, an endoscopic retrograde cholangiopancreatography was performed demonstrating leak of contrast in the genu of the pancreas. Pancreatic sphincterotomy was done followed by pancreatic duct stenting (Figure 7 and 8). Necrosectomy was repeated after a week, and the Axios stent was removed and replaced with a double-pigtail plastic stent for long-term drainage in view of ductal disruption. Both the cystogastrostomy stent and the pancreatic duct stent were removed at 3 months once the collections were completely resolved.

## DISCUSSION

Hepatic hemangiomas are benign vascular lesions that are believed to have either arisen from congenital hamartomas or from the dilation of existing blood vessels.^5^ Hepatic hemangiomas are the most common benign hepatic tumors with a prevalence of 0.4%–20% identified during autopsies.^5^ Symptomatology depends on the size of the lesion. Treatment for asymptomatic patients is not required. However, interventions are usually reserved in cases where the lesion grows >10 cm or reports of symptomatic recurrent compressive pain. Symptomatic patients can be managed surgically or with other nonsurgical modalities such as transcatheter arterial embolization (TAE) or radiofrequency ablation.^5,6^

The incidence of acute pancreatitis (AP) after liver embolization is 1.7%.^2^ The pancreas has a rich vascular supply. The pancreatic head receives blood from the superior pancreaticoduodenal artery, which is a branch of the gastroduodenal artery and the inferior pancreaticoduodenal artery, which originates from the superior mesenteric artery. The body and tail of the pancreas are supplied by the smaller branches of the splenic artery.^1^ Owing to this extensive vascular supply, complications after embolization are uncommon.

The exact mechanisms of how AP occurs after liver embolization are uncertain. However, some theorized reasons for AP after liver embolization are the possibility of reflux embolization.^1,4,6,7^ When selective embolization of hepatic artery is performed, reflux in the superior mesenteric artery is uncommon, minimizing the risk of ischemic changes in certain regions such as the pancreatic body and tail. However, when nontarget embolization is performed, areas such as the pancreatic head and uncinate process are more exposed to reflux embolization and, hence, to the development of ischemic changes ^1^ due to the pancreatic head and uncinate process, areas being perfused by small branches of the gastroduodenal artery.^3^ AP maybe the result of impairment in the arterial supply.^3,8^ An experimental rat model conducted by Redha et al found that embolization and complete occlusion of the splenic artery using polystyrene microspheres caused acute hemorrhagic pancreatitis in 100% of the cases.^3,8^ We theorize that in the patient mentioned in this case report, the likely mechanism of the development of necrotizing pancreatitis was from reflux embolization terminal arteries supplying the vulnerable areas of the pancreas. A previous study has also shown that AP was significantly less likely to occur in patients who received 2 mL or less of embospheres for an embolization procedure compared with patients who had received >2 mL.^1,3,6^ Larger volumes administered led to increased stasis in the target artery and thus increased likelihood of reflux of embolic material with resultant nontarget embolization.^6^ The onset of AP also helps determine the etiology, that is, within 24 hours would be ischemia due to the embospheres or several days later due to the effect of chemotherapy.^9^ Since AP can clinically mimic a postembolization syndrome, careful monitoring of serum pancreatic enzymes would be advisable in cases of abdominal pain. Obtaining a good arteriogram and identification of anatomical variations and arteriovenous shunts can help avoid most complications.^2,6^ TAE frequently causes pancreatic tissue damage, and the position of the inserted catheter tip is very important to avoid the pancreatic tissue damage by TAE.^7,10,11^

It is important to keep in mind complications such as AP when counseling patients about liver embolization procedures and when patients present with abdominal pain postembolization to ensure high quality care.

## DISCLOSURES

Author contributions: Ayesha Khan, J. Hawes, and Ahmed Khan: writing, revising/editing, and obtained images. J. Zhang: revising/editing. K. Szauter: revising/editing and final approval. M. Falamaki: final approval and contributed radiology images. ML Kueht and L. Cicalese: final approval. S. Parupudi: final approval and revising/editing.

Financial disclosure: None to report.

Previous presentation: The case was presented at the 2023 ACG Annual Scientific Meeting; October 23, 2023; Vancouver, BC, Canada.

Informed consent could not be obtained for this case report. All identifying information has been removed.
